# Benchmarking bacterial genome-wide association study methods using simulated genomes and phenotypes

**DOI:** 10.1099/mgen.0.000337

**Published:** 2020-02-25

**Authors:** Morteza M. Saber, B. Jesse Shapiro

**Affiliations:** ^1^​ Département de Sciences Biologiques, Université de Montréal, Montréal, QC, Canada

**Keywords:** GWAS, bacteria, simulation, linkage disequilibrium, benchmarking, power

## Abstract

Genome-wide association studies (GWASs) have the potential to reveal the genetics of microbial phenotypes such as antibiotic resistance and virulence. Capitalizing on the growing wealth of bacterial sequence data, microbial GWAS methods aim to identify causal genetic variants while ignoring spurious associations. Bacteria reproduce clonally, leading to strong population structure and genome-wide linkage, making it challenging to separate true ‘hits’ (i.e. mutations that cause a phenotype) from non-causal linked mutations. GWAS methods attempt to correct for population structure in different ways, but their performance has not yet been systematically and comprehensively evaluated under a range of evolutionary scenarios. Here, we developed a bacterial GWAS simulator (BacGWASim) to generate bacterial genomes with varying rates of mutation, recombination and other evolutionary parameters, along with a subset of causal mutations underlying a phenotype of interest. We assessed the performance (recall and precision) of three widely used single-locus GWAS approaches (cluster-based, dimensionality-reduction and linear mixed models, implemented in plink, pyseer and gemma) and one relatively new multi-locus model implemented in pyseer, across a range of simulated sample sizes, recombination rates and causal mutation effect sizes. As expected, all methods performed better with larger sample sizes and effect sizes. The performance of clustering and dimensionality reduction approaches to correct for population structure were considerably variable according to the choice of parameters. Notably, the multi-locus elastic net (lasso) approach was consistently amongst the highest-performing methods, and had the highest power in detecting causal variants with both low and high effect sizes. Most methods reached the level of good performance (recall >0.75) for identifying causal mutations of strong effect size [log odds ratio (OR) ≥2] with a sample size of 2000 genomes. However, only elastic nets reached the level of reasonable performance (recall=0.35) for detecting markers with weaker effects (log OR ~1) in smaller samples. Elastic nets also showed superior precision and recall in controlling for genome-wide linkage, relative to single-locus models. However, all methods performed relatively poorly on highly clonal (low-recombining) genomes, suggesting room for improvement in method development. These findings show the potential for multi-locus models to improve bacterial GWAS performance. BacGWASim code and simulated data are publicly available to enable further comparisons and benchmarking of new methods.

## Data Summary

1. Genomes used for measuring linkage disequilibrium (LD).

*Mycobacterium *: 3295 samples susceptible to pyrazinamide, downloaded from ftp://ftp.patricbrc.org/AMR_genome_sets/Mycobacterium/pyrazinamide/Susceptible.

*Escherichia *: 1582 samples susceptible and resistant to gentamicin, downloaded from ftp://ftp.patricbrc.org/AMR_genome_sets/Escherichia/gentamicin.

*Streptococcus *: 2169 samples resistant to trimethoprim, downloaded from ftp://ftp.patricbrc.org/AMR_genome_sets/Streptococcus/trimethoprim/sulfamethoxazole/Resistant.

2. Simulation datasets.

Simulation dataset for sample size 400: https://figshare.com/articles/bacterial_GWAS_benchmark_simulations_Sample_size_400/9956420.

Simulation dataset for sample size 700: https://figshare.com/articles/bacterial_GWAS_benchmark_simulations_Sample_size_700/9956426.

Simulation dataset for sample size 1000: https://figshare.com/articles/bacterial_GWAS_benchmark_simulations_Sample_size_1000/9956429.

Simulation dataset for sample size 2000: https://figshare.com/articles/bacterial_GWAS_benchmark_simulations_Sample_size_2000/9956441.

Simulation dataset for sample size 3000 and low LD: https://figshare.com/articles/bacterial_GWAS_benchmark_simulations_lowLD_Sample_size_3000/9956444.

Simulation dataset for moderate LD simulations: https://figshare.com/articles/bacterial_GWAS_benchmark_simulations_Medium_LD_dataset/9956456.

Simulation dataset for high LD simulations: https://figshare.com/articles/bacterial_GWAS_benchmark_simulations_High_LD_dataset/9956477.

**Impact Statement**

Microbial populations contain measurable phenotypic differences with important clinical and environmental consequences, such as antibiotic resistance, virulence, host preference and transmissibility. A major challenge is to discover the genes and mutations in bacterial genomes that control these phenotypes. Bacterial genome-wide association studies (GWASs) are families of methods to statistically associate phenotypes with genotypes, such as point mutations and other variants across the genome. However, compared to sexual organisms such as humans, bacteria reproduce clonally meaning that causal mutations tend to be strongly linked to other mutations on the same chromosome. This genome-wide linkage makes it challenging to statistically separate causal mutations from non-causal false-positive associations. Several GWAS methods are currently available, but it is not clear which is the most powerful and accurate for bacteria. To systematically evaluate these methods, we developed BacGWASim, a computational pipeline to simulate the evolution of bacterial genomes and phenotypes. Using simulated genomes, we found that GWAS methods varied widely in their performance. In general, causal mutations of strong effect (e.g. those under strong selection for antibiotic resistance) could be easily identified with relatively small samples sizes of around 1000 genomes, but more complex phenotypes controlled by mutations of weaker effect required 3000 genomes or more. We found that the elastic net (lasso) approach, a method that has only recently been applied to the GWAS problem, was particularly good at identifying causal mutations in highly clonal populations, with strong linkage between mutations – but there is still room for improvement. The BacGWASim computer code is publicly available to enable further comparisons and benchmarking of new methods.

## Introduction

Recent progress in sequencing technologies and consequently the rapid expansion of bacterial genomic data repositories have provided enormous opportunities to identify the genomic elements underlying clinically, environmentally and industrially important bacterial phenotypes and their evolutionary responses to changing environmental circumstances. Such discoveries could immensely improve our knowledge of the molecular mechanisms of important microbial phenotypes such as antibiotic resistance and virulence; thus, contributing to the development of new drugs, vaccines and antibiotics.

By identifying statistical associations between genotype and phenotype, genome-wide association studies (GWASs) can be used to dissect the genetic components of any measurable and heritable phenotype in an unbiased hypothesis-free manner. In humans, GWASs have been used to investigate genotype–phenotype association since the early 2000s, leading to the discovery of more than 149 000 trait-associated genomic markers, and in the past 5 years, the number of GWAS publications has increased by more than 300 % (1961 to 7796) [1]. An early application of a GWAS approach to bacteria attempted to unravel the genomic elements responsible for transforming harmless *Neisseria meningitidis* into a lethal pathogen causing cerebrospinal meningitis using multilocus sequence typing [2]. The difficulties and limitations of bacterial GWASs imposed by population structure were appreciated soon thereafter [3]. Nevertheless, over the past decade, GWASs applied to SNPs and k*-*mers (i.e. DNA words of length *k*) in microbial genomes have identified mutations and genes associated with antibiotic resistance [4–10], cancer [11], virulence [2, 12, 13] and host preference [14]. In contrast to human GWAS, however, bacterial association mapping is technically challenging due to the unique characteristics of bacterial populations, and the optimality of current approaches has yet to be established.

The objective of a GWAS method is to maximize statistical precision and power, in order to identify true causal genomic elements, while ignoring spurious associations. To do so, GWAS methods must overcome confounding factors, which are particularly acute in bacterial populations. The two main confounding elements in bacteria are genome-wide linkage disequilibrium (LD) interrupted by homologous recombination tracts, and strong population structure resulting from clonal expansions.

Genome-wide LD leads to type I errors (false positives) in GWAS tests, because linked non-causal mutations may hitchhike on the same genomic background (‘clonal frame’) as a causal mutation. A naïve GWAS approach will find the entire set of linked mutations to be associated with the phenotype. In bacterial species such as *Mycobacterium tuberculosis* that are virtually non-recombining, most of the genome is in complete LD, posing a major risk of type I error. In humans and other sexual species, LD is broken down by homologous recombination every generation, allowing GWAS tests to map the causal variants to a small genomic region. In bacteria, LD may span the entire genome, complicating fine mapping of causal variants. Even in bacterial species with relatively high rates of homologous recombination (e.g*. Streptococcus pneumoniae*), LD may still extend across the entire chromosome [15].

Population structure refers to a situation in which subpopulations have systematic differences in allele and phenotype frequencies [16, 17]. This can result in spurious associations between genotypes and phenotypes due to shared ancestry rather than causal associations. To control for the confounding effect of population structure, microbial GWAS tools have adapted single-locus approaches already used in human GWASs, including cluster-based techniques [6, 12, 18], dimensionality reduction [19–23], linear mixed models (LMMs) [4, 8, 10, 24, 25] and, recently, artificial intelligence using multi-locus models [26]. Here, we define single-locus approaches as models testing the association between a phenotype and a single variant at a time, repeated for all variants across the genome. In contrast, multi-locus models (including several machine-learning approaches) are fitted to the entire dataset at once. Although each of these approaches has been successful to varying extents, population structure is still a challenge in microbial GWASs and no gold standard solution has been established.

The power of a GWAS to identify causal variants underlying a phenotype is influenced by several other factors, including the sample size and the distribution of effect sizes. In human GWASs, most of the detected causal variants underlying complex phenotypes have an odds ratio (OR) <1.5 [27] due to the polygenicity of the traits and the fact that many human phenotypes of interest are disease traits that have been largely shaped by neutral evolution rather than strong selection. In bacteria, however, many phenotypes of interest, such as antibiotic resistance or host association, tend to be shaped by recent positive selection. Therefore, the genomic elements controlling bacterial traits are expected to have larger effect sizes [18]. For example, mutations conferring antibiotic resistance in *M. tuberculosis* [28] and *S. pneumoniae* [6] tend to have large effect sizes (OR >10). However, it has yet to be investigated whether smaller effect sizes are detectable and, if so, by which methods.

To determine best practices for microbial GWAS, it is essential to compare current GWAS methods in terms of their performance across a range of realistic effect sizes, recombination rates and sample sizes. For this purpose, here, we have developed a simulation platform called BacGWASim, which simulates bacterial genomes along a defined phylogenetic tree to capture mutation and recombination events in a clonal population structure. BacGWASim is tunable for a variety of evolutionary parameters in order to simulate a wide range of bacterial species. It then simulates bacterial phenotypes based on adjustable values of heritability, number of causal variants and their effect sizes. The major different classes of GWAS methods currently in use were then evaluated in terms of their precision [true positives/(true positives+false positives)] and power [true positives/[true positives+false negatives)] to identify true causal variants in the simulated bacterial populations. As an additional performance metric, we used the F1 score, defined as the harmonic mean of precision and power, which has been used elsewhere to evaluate the performance of GWAS tools [16]. These metrics are informative for the GWAS scenario in which there are relatively few true positives. We focused mainly on the effects of sample size, causal variant effect size, LD (recombination rates), while other parameters were kept constant. Notably, we held the mutation rate constant in order to compare across GWAS methods. In practice, mutation rate is an important parameter in determining GWAS power (e.g. if there is very little genetic diversity, it is unlikely that many GWAS hits will be found). In summary, we provide an extensible framework for simulating the evolution of bacterial genotypes and phenotypes, and for benchmarking new GWAS approaches as they become available.

## Methods

### Overview of the bacterial GWAS simulator (BacGWASim)

**Fig. 1.**
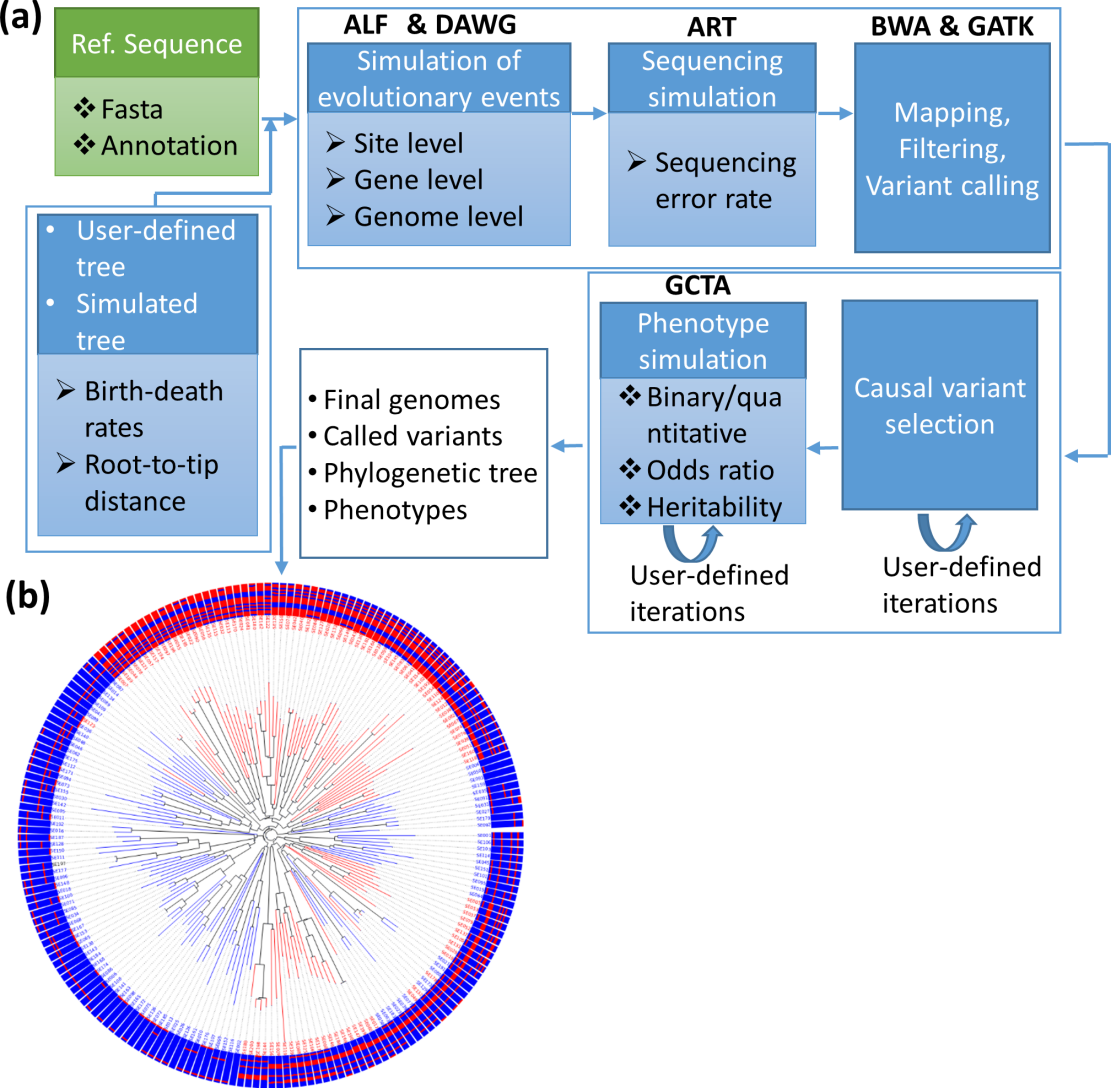
Overview of the bacterial GWAS simulation (BacGWASim) pipeline. (a) Taking a bacterial genome and annotations as input, evolutionary events are simulated across the branches of the phylogenetic tree (either user-defined or simulated based on a birth–death model), producing the final genomes and simulated markers as output. A phenotype is then assigned to each simulated sample based on the presence/absence of a set of causal SNPs. (b) Binary phenotypic states, shown as red or blue branches, mostly cluster in monophyletic subpopulations of closely related individuals due to population structure. The external rings denote the allele (red or blue) at each of the simulated causal loci (shown as concentric rings).

BacGWASim was developed with the goal of simulating a range of evolutionary parameters that can potentially confound microbial GWASs. In this first release, we primarily focused on simulating population structure and genome-wide LD, and evaluating GWAS methods to identify SNPs underlying a phenotype. BacGWASim starts with a bacterial whole genome and its annotations, and simulates a population across a pre-defined or simulated phylogenetic tree (Fig. 1a). A binary or continuous phenotype is then assigned to each genome in the population based on the emergence and evolution of a randomly chosen set of causal variants with a user-defined range of effect sizes. BacGWASim simulates bacterial genotypes and phenotypes in three main steps: (i) generating a phylogenetic tree, (ii) evolving genomes along the phylogenetic tree and (iii) simulating phenotypes.

### Generating the phylogenetic tree

A neutral model of speciation-extinction developed by Genhard [29] implemented in Artificial Life Framework (alf) [30] was used for the simulation of phylogenetic trees. This model allows for simulation of bacterial populations across a wide range of birth rates (λ), death rates (µ) and branch length distributions. A custom phylogenetic tree can also be provided by the user to provide the option for simulation of scenarios inferred from real bacterial populations.

### Simulation of genome evolution

For simulation of realistic genomic content, BacGWASim accepts any bacterial whole genome and the corresponding feature annotations as starting input. The elements of the genome are then extracted and classified in two groups of protein-encoding genes and intergenic regions (defined as sequence not annotated as a coding sequence). These two categories are often under distinct evolutionary constraints and their evolution is best captured by different evolutionary parameters. alf was used for the simulation of protein-encoding genes and dawg v.2 for the intergenic regions [31]. These implementations allowed for simulation of three categories of genomic events: (i) site-level events including codon and nucleotide substitution rates, insertion and deletion rates, and rate variation across sites; (ii) gene-level events including gene deletion, duplication and gene fission/fusion; and (iii) genome-level events including inversion, translocation and recombination through horizontal gene transfer. Equal nucleotide diversities across all evolutionary scenarios were set by fixing the mutRate parameter to 0.0004 in the alf phylogenetic tree simulator in order to remove confounding effects of differences in genetic diversity on GWAS. The resulting sequences of coding and intergenic regions at the tips of phylogenetic tree are then combined, while accounting for gene loss and transfer. Synthetic sequencing reads with Illumina-specific sequencing errors were generated for each simulated sample using the art next-generation sequencing read simulator [32]. The empirically measured error rates for Illumina paired-end sequencing estimated by Huang *et al*. [33] were used. These synthetic reads were then mapped to the reference genome using bwa [34] with default setting and the simulated variants were called using GATK Haplotype caller by setting ‘ploidy’ to 1 [35].

### Phenotype simulation

After genome simulation, a binary or continuous phenotype was then simulated for each member of the population based on an additive genetic model (equation 1) implemented in gcta [36]:

$y_{j}=sum\left(\frac{\left(x_{ij}-2p_{i}\right)}{\sqrt{2p_{i}\left(1-p_{i}\right)}}\times u_{i}\right)+e_{j}$(1)

where *y_j_* is the phenotype liability of individual *j, x_ij_* is number of reference alleles for the *i*th causal variant of the *j*th individual, *p_i_* is the frequency of the *i*th causal variant, *u_i_* is the allelic effect of the *i*th causal variant, and *e_j_* is the residual effect generated from a normal distribution with mean of 0 and variance of var ($sum\left(\frac{{(x}_{ij}-2p_{i})}{\sqrt{{2p}_{i}(1-p_{i})}}\times u_{i}\right)$) (1/*h^2^* - 1), where *h^2^* is the heritability of the phenotype defined by the user. Cases were sampled from the individuals with phenotype liabilities (*y*) exceeding the threshold of a normal distribution truncating the proportion of *K* (phenotype prevalence defined by the user), and controls were sampled from the remaining individuals.

A user-defined number of causal variants, minor allele frequency cut-off and range of effect sizes in units of OR are used to randomly select a set of ‘true’ causal variants from the pool of simulated markers. The phenotype labels are then simulated according to the presence/absence of the causal variants in each genome, user-defined values of heritability and prevalence of the desired phenotype. Across all the simulations in this paper, the ratio of case to control was set to 1 (simu-cc=population size/2−population-size/2), heritability was set to 1 (simu-hsq=1) and phenotype prevalence (or ‘disease prevalence’) was set to 0.5 (simu-k=0.5).

### Benchmark datasets and methods of bacterial GWAS

Bacterial genomes were simulated within ranges of three features of interest, keeping other parameters constant. (i) Sample size: bacterial populations with a range of 400 to 3000 sampled genomes were simulated to evaluate the effect of sample size on GWAS. (ii) Recombination rate: recombination rates in highly recombining *S. pneumoniae* estimated by Chewapreecha *et al*. [37] (mean ρ/θ≈0.20) were used for simulation of highly recombining populations. Moderate- and low-recombining populations were respectively simulated with ρ/θ ratios of 0.1 and 0.001. alf accepts the recombination rate (ρ/θ) via the lgtRate parameter. The maximum recombination tract length was set to the length of each gene considered. To simulate homologous recombination, the orthRep parameter was set to 1 in all scenarios. (iii) Effect size distribution: 18 causal markers with ORs (effect sizes) of 2, 3, 4, 7, 10, 11, 15 and 20 (natural logarithm in the range of 1 to 3) with minor allele frequency >0.1 were randomly chosen for phenotype simulation. The LD values between the selected markers were measured using bcftools [38] and markers with r^2^ >0.6 were discarded. This filtering step to remove strongly linked causal markers was included to ensure that these markers (including those of different effect sizes) were identifiable in the simulated datasets. We note that causal markers could still be linked to non-causal alleles, posing a challenge for GWAS methods to correctly identify the causal variant.

**Fig. 2.**
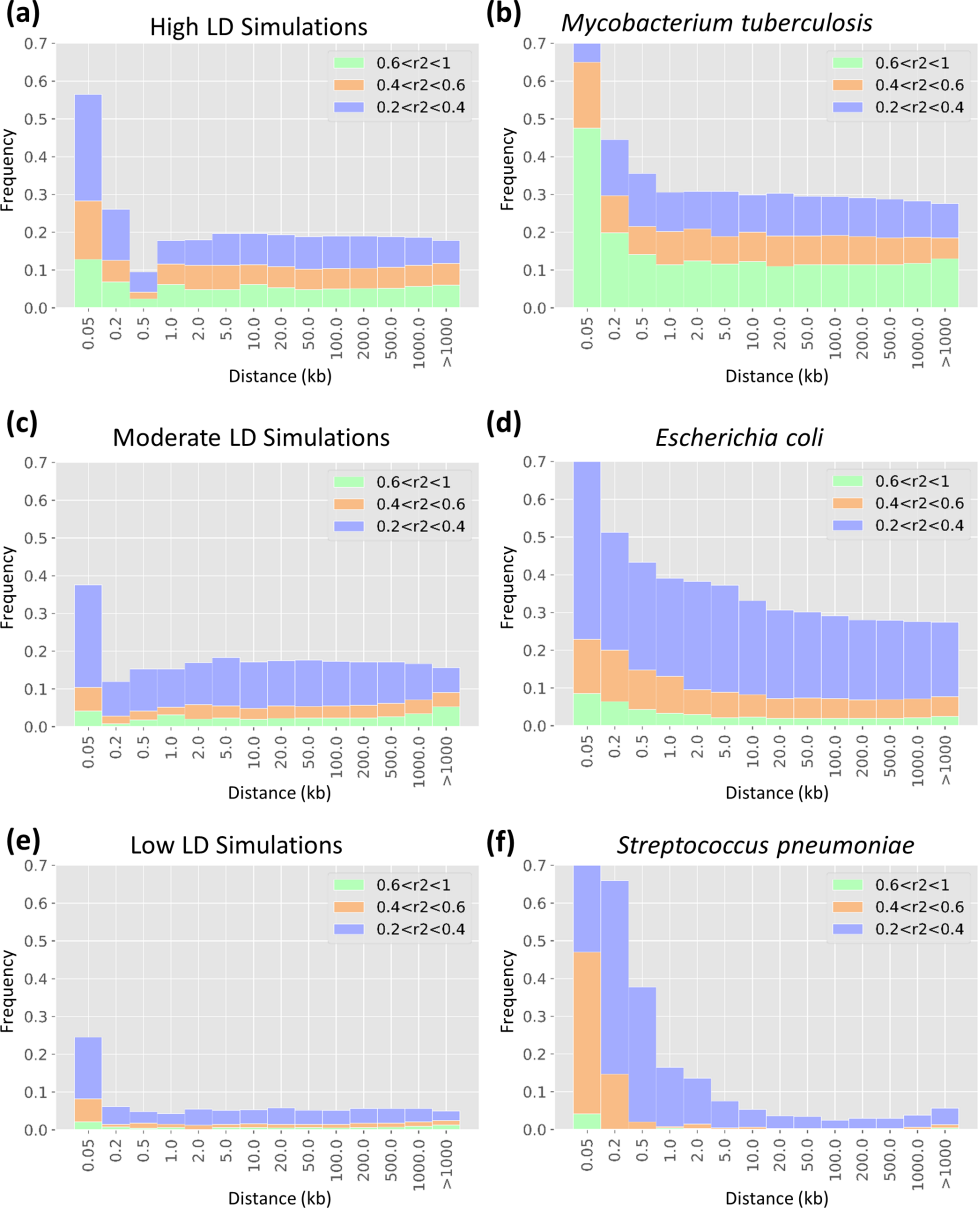
Landscape of genome-wide LD in bacterial species compared to BacGWASim simulations. The distribution of linkage, measured as r^2^ scores binned into three categories, is shown as a function of distance in the genome, in kb, within genomes of simulated (left panels) and real (right panels) bacteria.

To measure the range of LD in real bacterial species, genome data were retrieved from the card database [39], SNPs were called using Snippy [40] and linkage levels were measured in 1000 markers using Haploview [26] (Fig. 2). In every set of simulations, 100 000 randomly selected markers with minor allele frequency >0.01 were retained for GWAS analysis. An equal number of markers was selected in each simulation to make them comparable without any confounding effect of multiple testing across replicates. For each genome simulation, ten sets of randomly chosen markers were then used to generate ten replicate phenotype simulations. Using the called variants and phenotype labels as benchmark datasets, we then used the following methods to perform GWAS.

#### plink v1.9

This was employed using linkage agglomerative clustering based on pairwise identity-by-state (IBS) distances for population structure correction, and using Bonferroni corrections for multiple tests with the typically accepted genome-wide false-positive rate (FPR) of 0.05 [41].

#### seer 

This was implemented in pyseer v 1.2.0 with multidimensional scaling of pairwise k-mer based genetic distances to correct for population structure, and using a Bonferroni correction (at a genome-wide false discovery rate of 0.05) for the number of unique SNP patterns (i.e. only giving one count to a SNP with an identical presence/absence profile across genomes). In practice, however, the total number of patterns in our simulations were similar to the total number of SNPs (i.e. ~100 000). We also removed markers tagged with the errors ‘bad-chisq’, ‘pre-filtering-failed’, ‘lrt-filtering-failed’, ‘perfectly-separable-data’, ‘firth-fail’ and ‘matrix-inversionerror’ after the analysis [19, 42]. In our simulations, however, only two error types occurred, ‘bad-chisq’ and ‘high-bse’. These errors likely represent spurious associations; therefore, we removed them as recommended in the pyseer documentation.

#### FaST-LMM implemented in pyseer 1.2.0 using pairwise variant-based distances

This was used to correct for population structure, using unique patterns to estimate significance threshold, and by removing the tagged markers mentioned above (as for seer) after the analysis [42, 43]. It should be noted that the results obtained from the pyseer implementation of FaST-LMM may not be exactly the same as the standalone FaST-LMM due to the added pre-processing and post-processing steps done in pyseer.

#### FaST-LMM implemented **in pyseer 1.2.0 using phylogeny-based patristic distances﻿**

This was used to correct for population structure, using unique patterns to estimate significance threshold, and by removing the tagged markers mentioned above after the analysis [42, 43].

#### gemma v.98

This was used with pairwise variant-based genetic distances to correct for population structure and setting the option ‘gk’ to 1 for generating the relationship matrix [44].

#### Elastic-net multi-locus model implemented in pyseer 1.2.0

This was used by setting the alpha value to 1, without sequence reweighting. By fitting an elastic net regression to the data, the top 75 % of markers with coefficients above zero were selected, and a *P* value for each selected marker was calculated using a chi-square test [45]. Bonferroni correction thresholds were determined based on the number of selected markers (ranging between 500 and 1000 across simulations). The threshold for genome-wide false discovery rate was set to 0.05. It should be noted that by setting the alpha value to 1 (as done here), the elastic net regression becomes equivalent to a lasso regression. By introducing more sparsity than ridge regression, lasso regressions are a suitable choice for identification of important features in high-dimensional datasets with many irrelevant features, especially cases where there are far more irrelevant dimensions than samples [46], which is the case for GWAS. In the pyseer documentation, the terms ‘univariate’ and ‘multivariate' are used to refer to single-locus and multi-locus models, respectively.

The performance of each GWAS method was assessed based on the mean values of precision, recall and F1 scores, and the corresponding sds across ten replicate simulations for each parameter combination, where: precision=true positives/(true positives+false positives), recall=true positives/(true positives+false negatives), and F1=2×(precision×recall)/(precision+recall). GWAS analysis can be considered as a classification task on a highly asymmetric dataset: in the case of our simulations, there were 99 984 true negative and only 16 true positives. Positive hits (whether true or false) were defined below a *P* value threshold of 0.05 after Bonferroni correction for 100 000 tests. Therefore, we used precision, recall and their harmonic mean (the F1 score) as our main performance metric, because they can handle this uneven class distribution. We also calculated the FPR (defined here as the number of false positives divided by the sum of false positives and true negatives).

## Results

### Simulating bacterial genomes and phenotypes

To systematically benchmark bacterial GWAS approaches, we first developed an appropriate simulator of bacterial genomes and phenotypes, BacGWASim (see Methods; Fig. 1a). The simulator starts from a real reference genome with gene annotations and then allows this genome to evolve along a user-defined or simulated phylogenetic tree capturing the population structure (Fig. 1b). Phenotypes are simulated according to a heritability function of causal SNPs, with user-defined effect sizes. Realistic sources of noise, such as sequencing error and read mapping, are also included. Although other evolutionary parameters (e.g. mutation rates) are tunable in the simulation, here we varied three key parameters with a likely effect on GWAS performance: sample size, recombination rate and effect size of causal mutations. We began with the genome sequence and phylogenetic tree of a well-studied species, *S. pneumoniae* (although the user could alternatively choose to simulate a phylogeny *de novo* using a birth–death process). A range of genome-wide LD was then simulated to approximate the range of LD observed in bacterial species with high, moderate and low recombination rates. While not an exact match, these simulations approximate the LD landscapes of *S. pneumoniae, Escherichia coli* and *M. tuberculosis *, respectively (Fig. 2). We will begin by focusing on the high-recombining, low LD simulations to explore the effects of sample and effect sizes on GWAS performance, and then revisit the effects of LD to conclude. In all simulations, phenotype heritability was set to one, with an equal number of cases and controls in each dataset. The BacGWASim codes are publicly available at: https://github.com/Morteza-M-Saber/BacGWASim.

### GWAS power to detect variants of large effect reaches a plateau at 3000 genomes

**Fig. 3.**
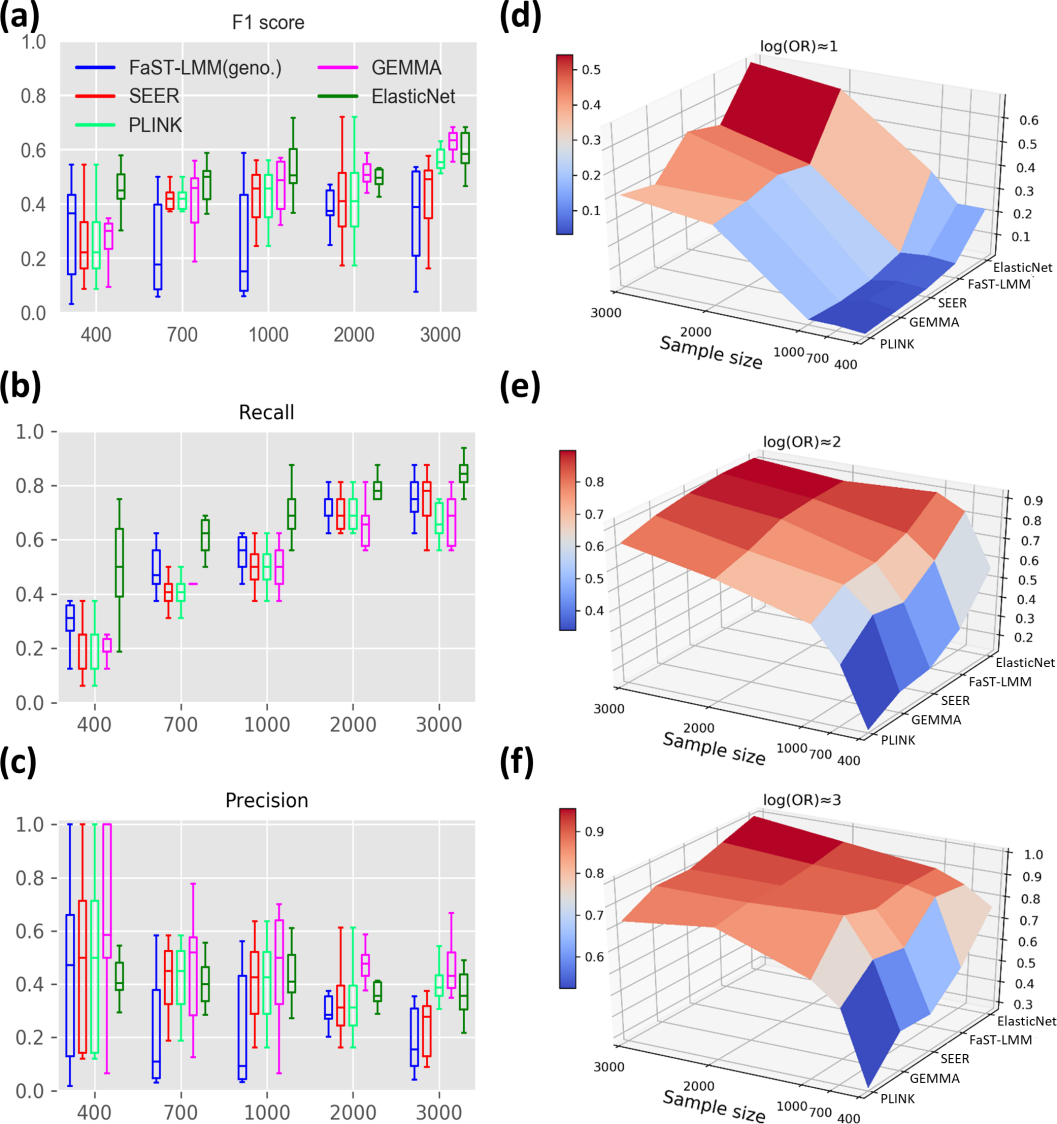
Performance of GWAS methods in simulations with low LD. (a, b, c) The boxplots on the left show the median and interquartile range, averaged across a range of effect sizes over ten replicates in each scenario (log OR=1 to 3). (a) F1 score of bacterial GWAS methods across a range of sample sizes under low LD. (b) Recall rates across the range of sample sizes. (c) Precision rates across the range of sample sizes. (d, e, f) The surface plots on the right show recall rates in separate categories of effect sizes.

**Fig. 4.**
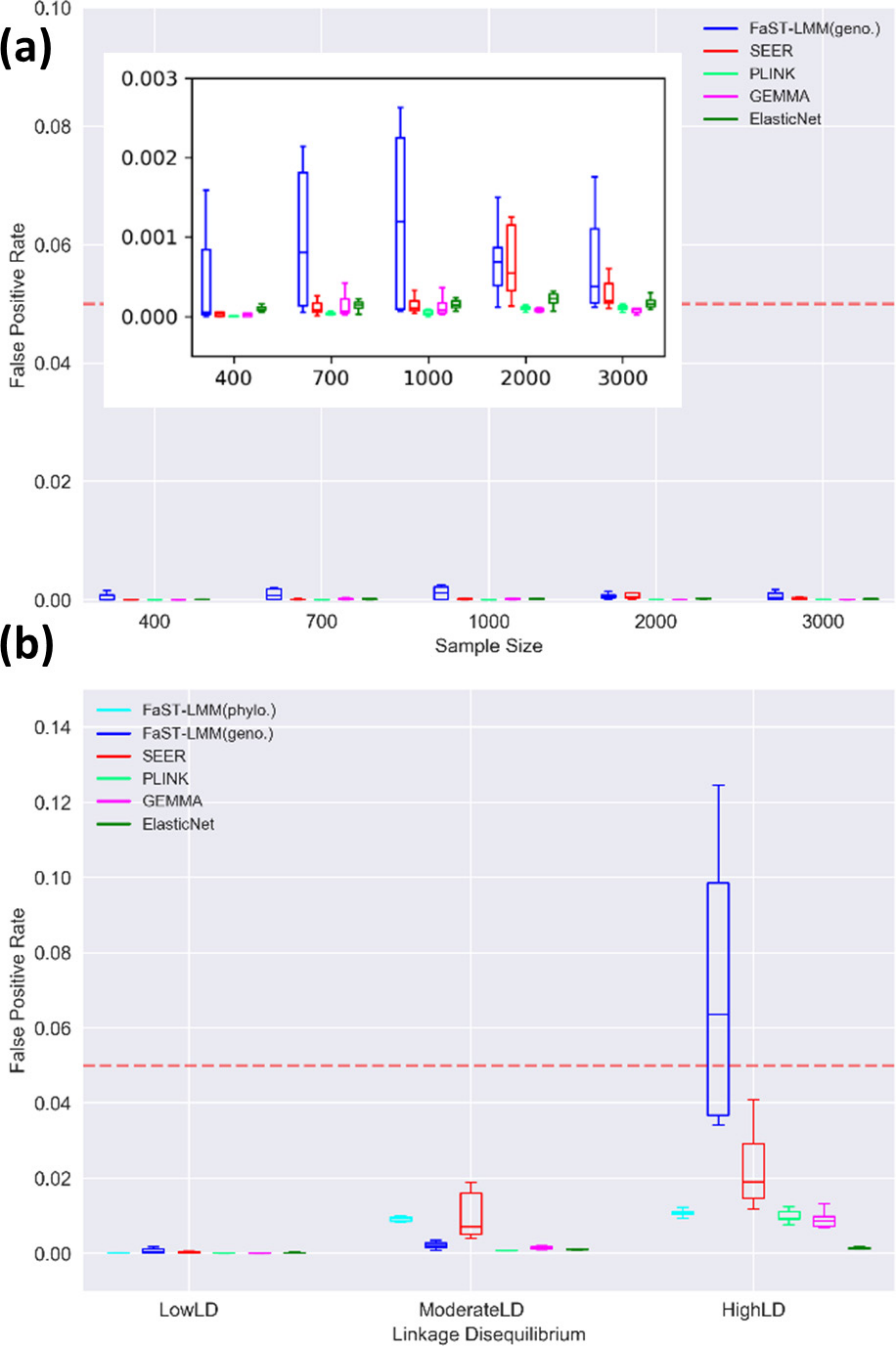
FPRs are generally acceptable across methods. FPRs are shown (a) across a range of sample sizes at low LD, and (b) across a range of LD in a sample size of 3000 genomes. The typical threshold of FPR=0.05 is displayed as a dotted red line. Note that the FPR is already corrected for multiple tests, since it is calculated based on the number of false positives and true negatives identified after Bonferroni correction (see Methods). The inset in panel (a) shows the same data as in the main image with an adjusted *y*-axis for better clarity. Boxplots show the median and interquartile range, averaged across a range of effect sizes over ten replicates in each scenario (log OR=1 to 3).

Early GWASs were able to identify causal variants of large effect with relatively small sample sizes (in the order of 100 genomes), likely because the phenotypes investigated were under strong positive selection [4, 9, 12, 14, 18]. However, recent advances in high-throughput sequencing technologies now make it feasible to sequence thousands of genomes. To evaluate the effect of increasing sample size on the power to detect causal mutations within a range of effect sizes, we measured the performance of bacterial GWAS methods on a range of sample sizes. In a high-recombining, low LD population (Fig. 2e), the elastic net multi-locus model (implemented in pyseer with alpha set to one, making it equivalent to lasso regression) was consistently amongst the methods with highest F1 scores across the range of sample sizes, with scores ranging between 0.44 and 0.60 (Fig. 3a), and precision in the range of 0.40 to 0.47 (Fig. 3c). The LMM implemented in gemma and clustering approach implemented in plink also showed low rates of false positives comparable to elastic nets (Fig. 3c); however, they had lower recall (Fig. 3b). FaST-LMM implemented in pyseer, despite its high recall (Fig. 3b), achieved lower F1 scores due to its relatively high number of false positives (Fig. 3c). With relatively small sample sizes (<1000), all methods except for elastic net showed poor performance in detecting causal variants with low effect sizes. Although all methods showed FPRs below the typical threshold of 0.05, there was some variation across methods (Fig. 4). Elastic nets outperformed other methods at moderate and high LD in particular (Fig. 4b).

A general pattern observed across all methods is that after a rapid surge in recall rate by increasing the sample size to 1000, the power improvement slows down and reaches a plateau around 3000 samples (Fig. 3b). Breaking down these comparisons by effect size shows that, as expected, causal markers with low effect size (log OR ≈1) are the most sensitive to sample size. The power to detect such variants is <0.1 in single-locus methods and <0.35 in elastic nets in samples sizes below 1000. Power improves to 0.37–0.48 for single-locus methods and 0.68 for elastic nets at a sample size of 3000 (Fig. 3d). In contrast, recall rate (power) for causal markers with higher effect sizes (log OR ≈2 and log OR ≈3) is more uniform across methods and reaches a maximum of 0.78 in single-locus models and 0.91 in elastic nets, with only 1000 genomes sampled. Beyond 1000 samples, the increase in recall tends to be small, reaching a plateau around 3000 samples for most methods (Fig. 3e, f) .

### Correcting for population structure

We next investigated how GWAS power varies across the four methods to correct for population structure: single-locus models including cluster-based approaches, dimensionality reduction and LMMs, and the multi-locus model using elastic nets. In single-locus models, the association between each of the markers (SNPs or k-mers) and the desired phenotype is investigated separately. Therefore, the covariance between the markers due to population structure needs to be included explicitly. Multi-locus models, however, include all the genetic variants at once, and by this means, covariance between the variants are implicitly included in the analysis. Our findings show that the multi-locus elastic net model has superior power relative to single-locus models in controlling for population structure and genome-wide LD, especially in small sample sizes (Fig. 3d, e, f) and when there is strong linkage across the genome, as discussed below. We consider each of the single-locus methods separately below.

### Cluster-based approaches

One of the classic methods for controlling the confounding effect of population structure is to identify clusters of related individuals within the overall population and then test for association conditional on these subpopulations. Subpopulations can be inferred using a variety of methods [41, 47–49] and then a weighted association test is performed for each genomic marker across the defined clusters (e.g. with the Cochran–Mantel–Haenszel test). The proportion of population structure captured in this approach, however, depends on the threshold used for clustering. Choosing a strict threshold for clustering will improve the precision of the test, but at the expense of reducing the recall score. To measure the effect of the choice of clustering threshold on the power of cluster-based methods, we performed linkage agglomerative clustering based on IBS distances implemented in plink across a range of thresholds. The threshold was defined as the maximum number of individuals allowed to group in one cluster. By relaxing the threshold, a larger number of samples with lower genetic similarity are included in each cluster; therefore, population structure will not be fully captured. In contrast, setting a very strict threshold will make the association test conservative by over-correcting for population structure.

**Fig. 5.**
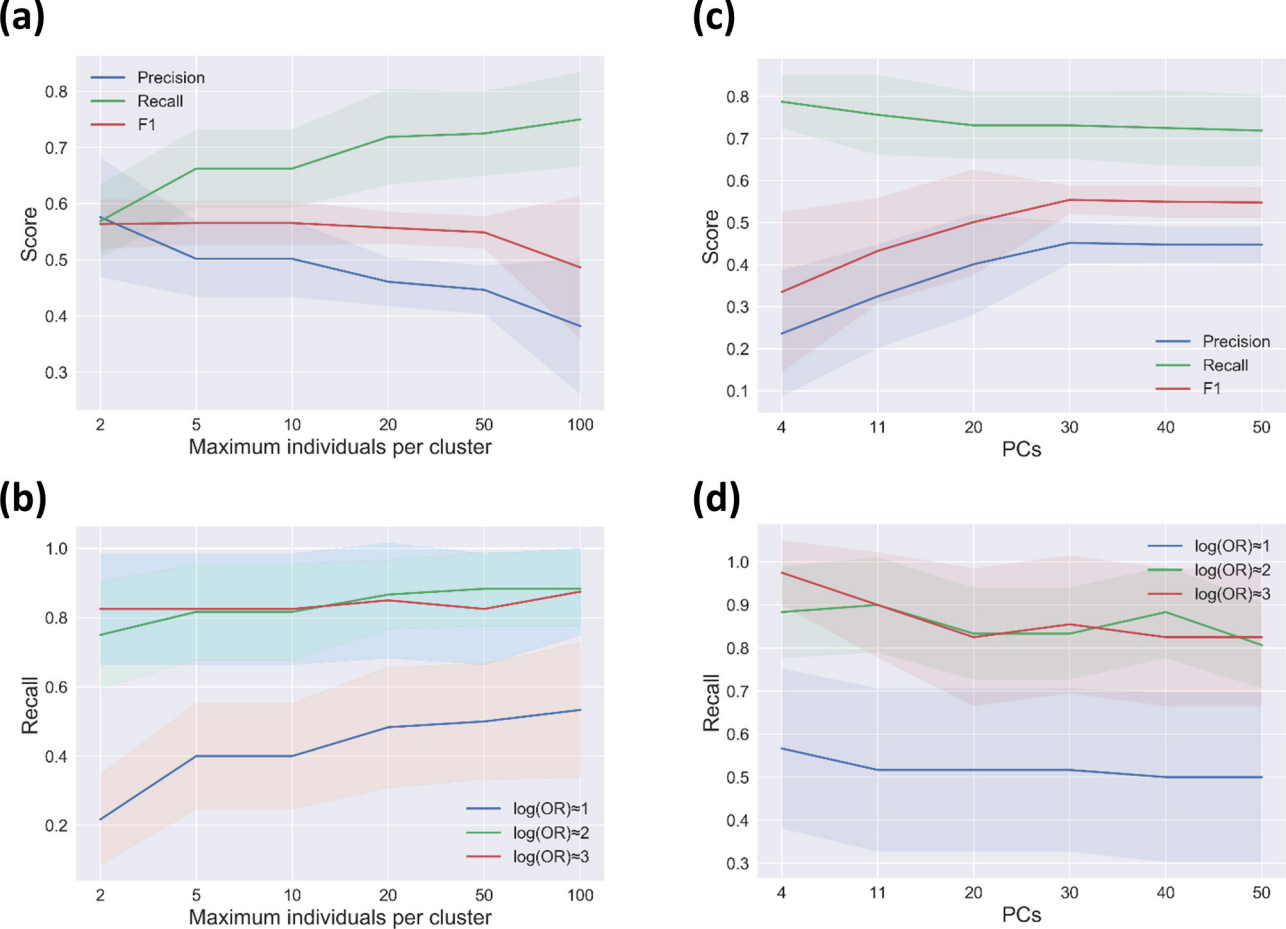
GWAS performance varies according to different levels of correction for population structure in a dataset of 3000 genomes simulated under low LD. (a) Effect of the clustering threshold in terms of the maximum number of individuals per cluster in the GWAS. (b) GWAS power (recall) across a range of clustering thresholds for identifying causal markers within separate categories of effect sizes. (c) Effect of variation in the number of included principal components (PCs) as covariates used for correction. (d) GWAS power (recall) across a range of included PCs for detecting causal markers within separate categories of effect sizes. Shaded regions represent ±1 sd.

Using simulated datasets of 3000 genomes with low LD (Fig. 2e), we varied the population structure correction from strict (maximum number of two individuals per cluster) to weak (up to a hundred individuals per cluster). As expected, going from strict to weak correction improves recall (from 0.56 to 0.75) at the expense of precision, which drops from 0.57 to 0.38 (Fig. 5a). The choice of clustering threshold significantly affects the ability to detect variants of low effect (log OR≈1), but has little effect on variants of larger effect (Fig. 5b). In the absence of established standards for choosing a clustering threshold, our results suggest that the choice may not matter for the detection of large-effect variants, but could significantly bias the detection of markers with low-effect sizes.

### Dimensionality reduction

In dimensionality reduction, a relatedness matrix of samples is projected onto a lower number of components, and then a certain number of these components are chosen as fixed-effect factors in the linear regression (or logistic regression in the case of binary phenotypes) to account for population structure. The two most popular and nearly identical methods for dimensionality reduction in GWASs are the principal component ANOVA-standardized relationship matrix based on SNP data [41] and the metric multidimensional scaling of genetic similarities based on pairwise distance matrix constructed using the shared k-mer content between all samples [19]. The former has been widely used in eukaryotic GWASs where there is low variation in pan-genome size, while the latter is specifically designed for bacterial GWASs and uses an alignment-free k-mer based approach to estimate genetic similarities based on core and accessory genome distances. Like cluster-based approaches, the proportion of population structure controlled in this approach depends on the number of components included as covariates in the regression analysis. Although there are some recommended methods for determining the optimal number of included components, such as visual estimation using the scree plot [42], the number of included components is subjective and is a matter of sensitivity–specificity trade-off. Including more components is likely to improve the precision of GWAS and reduce type I error caused by population structure, at the expense of decreasing the recall score.

To evaluate the effect of the number of included components in a dimensionality reduction-based bacterial GWAS, we tested the seer method implemented in pyseer on a simulated high-recombining dataset of 3000 samples (Fig. 2e). According to the scree plot, 4 or 11 are good choices for the number of components to be included in this particular analysis, as there are subtle but distinct drops after these number of dimensions (Fig. S1, available with the online version of this article). However, the F1 score reaches its maximum after inclusion of 30 dimensions (Fig. 5c). By increasing the number of components from 4 to 30, the precision significantly improved from 0.23 to 0.45, then reaches a plateau. Meanwhile, the recall score is relatively unaffected (dropping from 0.79 to 0.73), leading to total improvement of the F1 score (from 0.33 to 0.55) with an increasing number of components. As expected, recall is better for markers of high or medium effect, but recall does not vary much with increasing components for any category of effect size (Fig. 5d). In general, our results indicate that the choice of included components significantly affects the performance of dimensionality reduction-based stratification correction, and the lack of a standard protocol to identify the optimum number of included principal components can limit the application of this method.

### LMMs

The LMM is an extension of linear regression, which allows the inclusion of both fixed and random effects as covariates. By using a kinship matrix to model the variance of a random effect, LMMs consider the genetic relationships between all samples rather than selecting a proportion of the population structure (as in the cluster-based and dimensionality-reduction approaches described above) and has been shown to control type I error without loss of power compared to GWAS performed without population stratification correction [15]. gemma [44] and FaST-LMM (a factored spectrally transformed LMM, that is essentially an approximation to LMM) [43] are two popular LMM-based GWAS methods and have been used in recent bacterial GWASs, either as standalone methods, or as implemented in BugWAS [24], dbgwas [25] or pyseer [42].

**Fig. 6.**
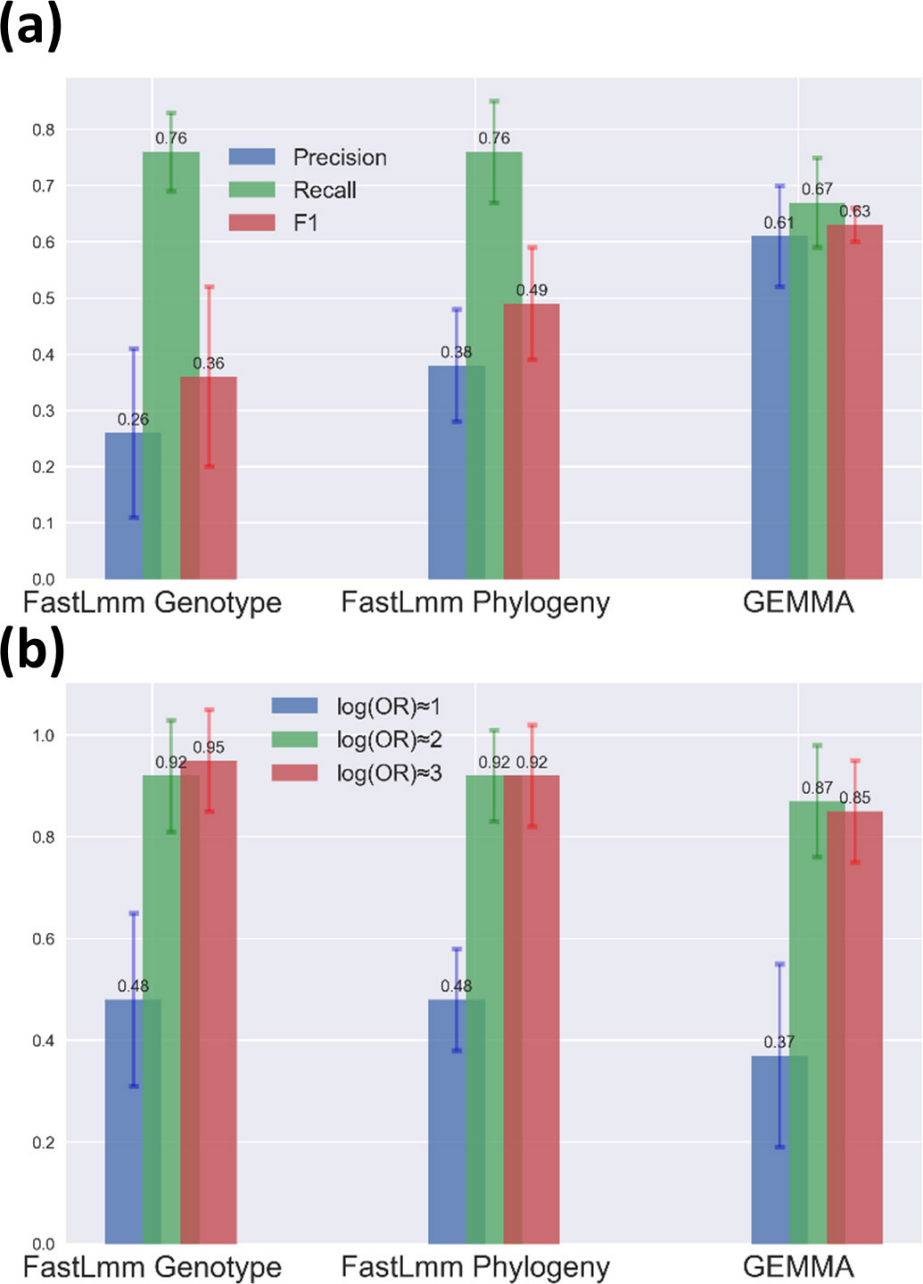
Performance of LMM-based GWAS methods in a dataset of 3000 simulated genomes under low LD. (a) Comparison of different implementations of LMMs in terms of precision, recall and F1 scores. (b) Comparison of the recall (power) of different implementations of LMMs in identifying causal markers within separate categories of effect sizes. Bar heights show the means across 10 replicate simulations and error bars show the sd.

To compare the power of LMM-based GWAS methods, we tested the performance of gemma and FaST-LMM implemented in pyseer on the same simulated high-recombining dataset of 3000 samples (Fig. 2e). pyseer provides the option to construct the kinship matrix using either variant-based genetic distances or by extracting patristic distances from the phylogeny, while gemma recommends using the centred genotype matrix. Averaged across effect sizes, gemma is the most efficient method to control for type I errors caused by population structure and results in the highest F1 score (Fig. 6a). In FaST-LMM, the phylogeny-based correction for population structure outperforms the genotype matrix, mainly due to a boost in precision (Fig. 6a). However, in this case the ‘true’ phylogeny (known from the simulation) was used, but must be estimated in real applications. Therefore, the accuracy of a phylogenetic correction might be lower depending on the choice of methods to construct the phylogenetic tree. FaST-LMM has slightly higher recall (power) than gemma, and its advantage was most pronounced for variants with low effect sizes (Fig. 6b). All methods had high and nearly equal power (>0.85) in identifying causal variants with high effect sizes (log OR ≈2 and log OR ≈3). However, FaST-LMM has higher power for detecting variants on the low end of the scale investigated here (log OR ≈1) (Fig. 6b).

### Current GWAS methods perform poorly under moderate to high LD

**Fig. 7.**
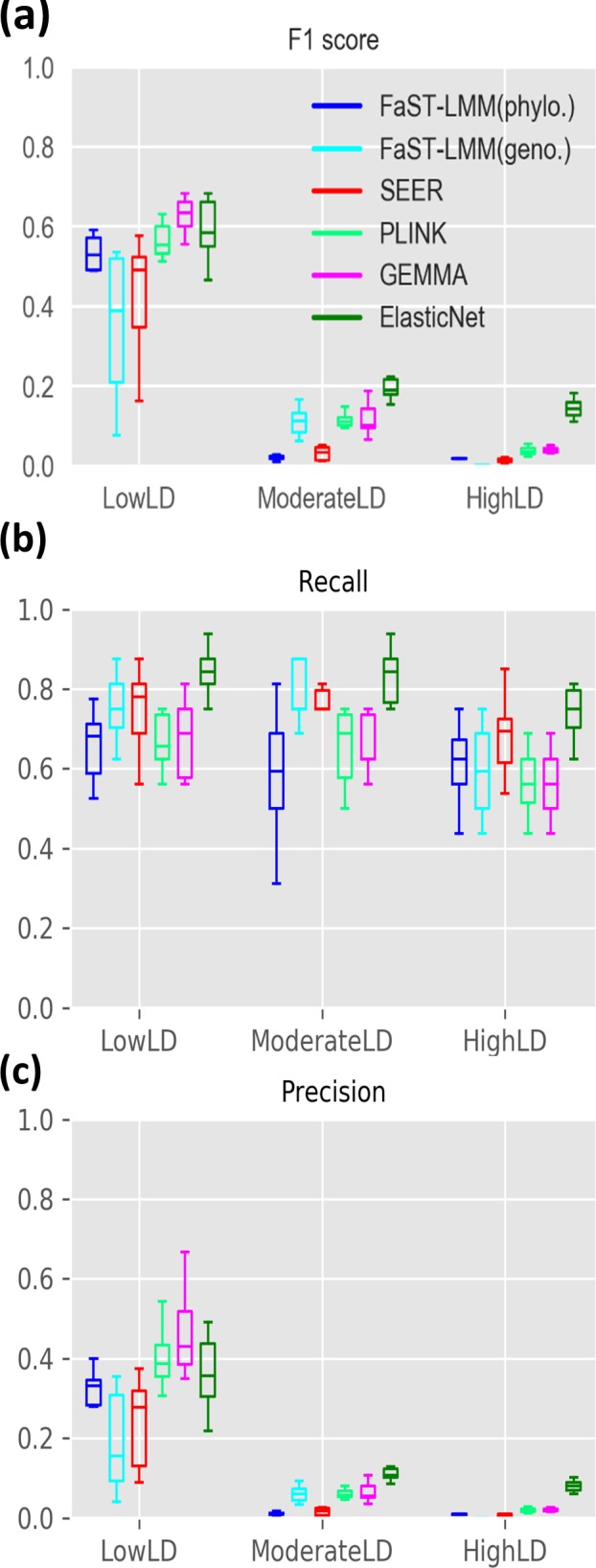
Effect of LD on GWAS performance. (a) F1 score, (b) recall and (c) precision of GWAS methods in datasets of 3000 genomes with low, moderate and high genome-wide LD levels. Boxplots show the median and interquartile range, averaged across a range of effect sizes over ten replicates in each scenario (log OR=1 to 3).

Higher genome-wide LD is expected to reduce the precision of association testing, because hitchhiking non-causal mutations are identified as false positives. To assess the influence of LD on GWAS performance, we tested each method across a range of simulated datasets with low, moderate or high genome-wide LD (Fig. 2). In general, the elastic net implemented in pyseer (with alpha values equivalent to lasso) considerably outperforms other methods at moderate or high LD (Fig. 7). At low LD, elastic nets perform similarly to gemma, the best single-locus method (Fig. 7a). At high LD, elastic nets achieve ∼75 % power, compared to ∼60 % in single-locus methods (Fig. 7b). All methods suffer a loss of precision with increasing LD, but elastic nets retain the highest precision at high LD (Fig. 7c). Still, the median precision of elastic nets at high LD is ∼8 %, suggesting significant room for improvement. The FaST-LMM approach using the genotype-matrix for population structure correction was most severely affected by LD, with precision dropping from 0.36 at low LD to <0.001 at high LD (Fig. 7c). However, FaST-LMM with the phylogeny-based correction showed comparable results to the best performing single-locus-model methods. Amongst the single-locus models, the mixed model approach implemented in gemma tended to perform somewhat better than others at moderate or high LD (Fig. 7a). Q-Q plots further indicate gemma to be the best performing method across the range of LD among the single-locus models (Fig. S2).

## Discussion

We developed a platform to simulate bacterial genomes and phenotypes based on the emergence and evolution of causal variants along a phylogenetic tree. The simulator is tunable for relevant evolutionary parameters. Although it was designed for benchmarking GWAS tools, it could also be used for other applications in bacterial population genomics and epidemiology. Here, we focused on how GWAS method performance is affected by sample size, recombination (LD) and causal variant effect sizes. We only considered point mutations in the core genome as causal variants, but this could be extended to causal variants in the pangenome (i.e. gene presence/absence), which can be identified with k-mer approaches [14] such as seer, BugWAS, dbgwas and others. Although a full exploration of core and pangenome associations is beyond the scope of the current work, we expect our major findings to hold when causal variants in the pangenome are included. Our evolutionary model is also ‘neutral’ in that it does not consider positive selection on causal variants (e.g. antibiotic-resistance mutations that have a selective advantage). Future versions of BacGWASim could explicitly model an increased replication rate (clonal expansion) of bacteria encoding causal variants, perhaps more realistically capturing the evolution of certain phenotypes.

Our results confirmed that currently popular GWAS methods perform poorly when applied to bacteria with relatively clonal population structures yielding moderate or high LD (e.g*. E. coli* or *M. tuberculosis *) [24]. In such clonal populations, fine-mapping of causal mutations may be impossible and identification of phenotype-associated lineages may be the best possible outcome [24]. More encouragingly, causal variants can be detected with relatively high power and precision in higher-recombining populations, akin to *S. pneumoniae *. These results highlight the importance of assessing the LD landscape of the target organisms before deciding on a sample size and GWAS design. It also suggests significant room for improvement in GWAS method development, particularly for highly clonal bacteria for which we are currently only able to detect lineage-specific associations [24]. Although we did not test homoplasy-based methods such as phyC [9] and treeWAS [16] due to their computational burden, especially with large sample sizes [15], they would be worth evaluating in the future. These methods could hold promise, at least for special cases where phenotypes are controlled by homoplastic mutations and an accurate phylogenetic tree can be inferred. Homoplasies do occur in our simulations (Fig. 1b), but their rate is not explicitly controlled and their impact, thus, is hard to assess. In the future, BacGWASim could be extended to explicitly model homoplastic mutations and to assess the performance of homoplasy-based methods.

Of the GWAS methods evaluated here, the elastic net multi-locus model (equivalent to lasso) generally performed best, followed by the mixed model approach implemented in gemma. The clustering approach implemented in plink also performed well, but varied significantly depending on the clustering threshold, which can be challenging to optimize. Elastic nets implemented in pyseer also provide the possibility to perform a GWAS on k-mers or unitigs, whereas this is not as easily implemented in gemma or plink. Although elastic nets had the highest precision of the methods tested, there is significant room for improvement, as mentioned above. However, particularly for highly clonal populations, there may be a limit to what can be learned from GWAS approaches, and some combination of experimental and observational studies may be required.

Our results also help explain the success of early bacterial GWASs with low sample sizes. We found that, in a high-recombining population, a sample size ~1000 is sufficient to reliably detect causal variants of strong effect (log OR>=2) with high power (>0.80). Such strong effect sizes may be common for antibiotic-resistance mutations, and other variants under strong positive selection. However, samples sizes >3000 will likely be needed to detect variants of lower effect (log OR ~1), which may be more common for more ‘complex’ phenotypes with lower heritability. Of course, the sample size required to achieve a desired power will vary depending on the recombination rate and population structure of the organism of interest. Thus, BacGWASim provides a tool for study-specific power calculations. Although other simulators of bacterial evolution are available, these tend not to model phenotype evolution, which is essential for benchmarking GWAS methods, nor do they provide as much control over genetic variation at the level of nucleotides, genes and genomes [50, 51]. We previously developed a power calculator for the special case of a homoplasy-based GWAS applied to a highly clonal bacterial population with a known phylogeny [52]. BacGWASim provides a much more general and flexible power calculator.

This work is somewhat limited in initial scope, as we focus on a subset of evolutionary parameters which we deem most relevant to GWAS benchmarking. Future studies could further explore variation in the mutation rate, causal allele frequencies, high LD among causal variants, case–controls ratios, environmental factors or selective pressures that differ between sub-populations. In an age of a rapidly growing array of options for performing GWASs, we hope that our results are instructive in quantifying general trends, and that our simulation platform can continue to be used to benchmark novel methods as they appear.

## Data bibliography

1. Genomes used for measuring LD in *Mycobacterium*: 3295 samples susceptible to pyrazinamide, downloaded from ftp://ftp.patricbrc.org/AMR_genome_sets/*Mycobacterium*/pyrazinamide/Susceptible (2019).

2. Genomes used for measuring LD in *Escherichia*: 1582 samples susceptible and resistant to gentamicin, downloaded from ftp://ftp.patricbrc.org/AMR_genome_sets/Escherichia/gentamicin (2019).

3. Genomes used for measuring LD in *Streptococcus*: 2169 samples resistant to trimethoprim, downloaded from ftp://ftp.patricbrc.org/AMR_genome_sets/Streptococcus/trimethoprim/sulfamethoxazole/Resistant (2019).

4. Saber, M. M., Shapiro, B. J., Simulation dataset for sample size 400 available at: https://figshare.com/articles/bacterial_GWAS_benchmark_simulations_Sample_size_400/995642 (2019).

5. Saber, M. M., Shapiro, B. J., Simulation dataset for sample size 700 available at: https://figshare.com/articles/bacterial_GWAS_benchmark_simulations_Sample_size_700/995642 (2019).

6. Saber, M. M., Shapiro, B. J., Simulation dataset for sample size 1000 available at: https://figshare.com/articles/bacterial_GWAS_benchmark_simulations_Sample_size_1000/9956429 (2019).

7. Saber, M. M., Shapiro, B. J.,Simulation dataset for sample size 2000 available at: https://figshare.com/articles/bacterial_GWAS_benchmark_simulations_Sample_size_2000/9956441 (2019).

8. Saber, M. M., Shapiro, B. J., Simulation dataset for sample size 3000 and low LD available at: https://figshare.com/articles/bacterial_GWAS_benchmark_simulations_lowLD_Sample_size_3000/9956444 (2019).

9. Saber, M. M., Shapiro, B. J., Simulation dataset for moderate LD simulations available at: https://figshare.com/articles/bacterial_GWAS_benchmark_simulations_Medium_LD_dataset/9956456 (2019).

10. Saber, M. M., Shapiro, B. J., Simulation dataset for high LD simulations available at: https://figshare.com/articles/bacterial_GWAS_benchmark_simulations_High_LD_dataset/995647 (2019).

## Supplementary Data

Supplementary material 1
