# Supplementary material for: Benchmarking bacterial genome-wide association study methods using simulated genomes and phenotypes
Source: Microb Genom. 2020 Feb 25;6(3):e000337. doi: 10.1099/mgen.0.000337 (PMC7200059; doi:10.1099/mgen.0.000337)
Supplement: Supplementary material 1 [file mgen-6-337-s001.pdf]

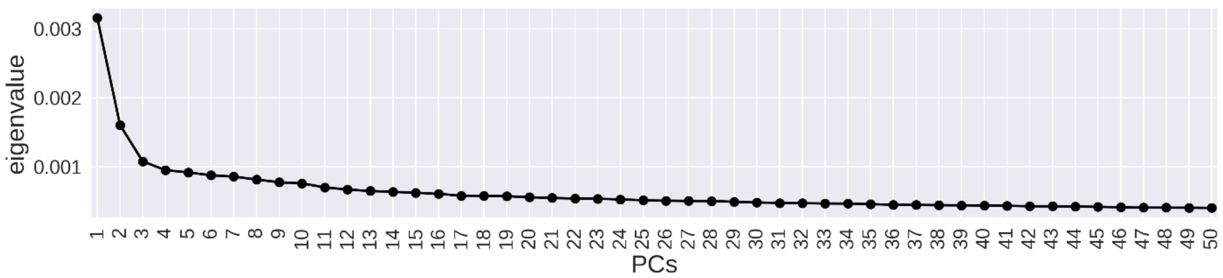

Figure S1. Scree plot of multi-dimensional scaling analysis.

There are major and minor drops after four and eleven dimensions indicating these values to be reasonable choices for the number of included components in the logistic regression analysis of GWAS. It must be noted that the optimal number of included dimensions inferred from scree plot is study-specific and can be different in each specific dataset.

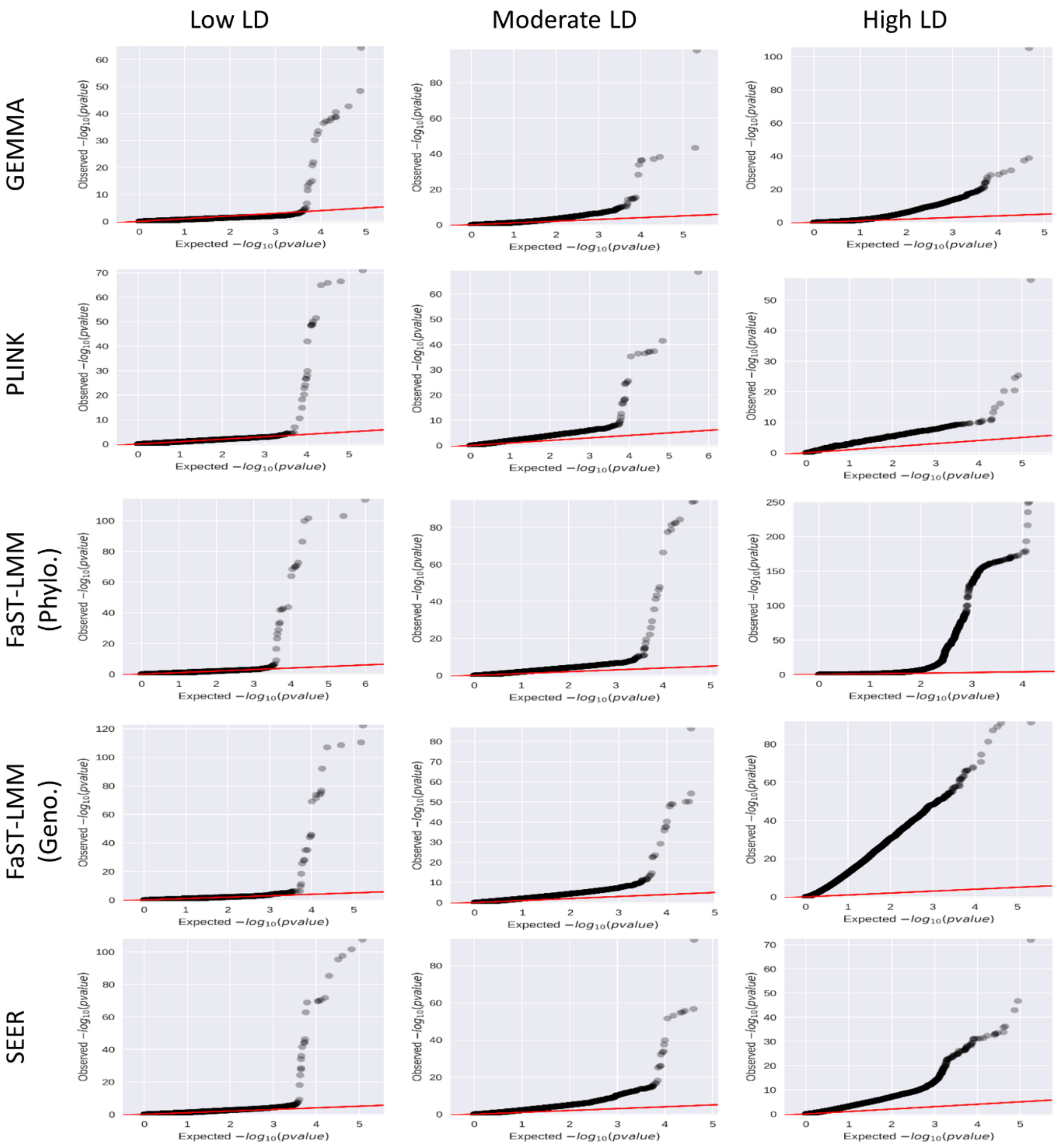

Figure S2.Q-Q plots of GWAS analysis using univariate models.

Q-Q plots of GWAS analysis in low, moderate and high LD datasets using GEMMA, Clustering approach in PLINK, FaST-LMM implemented in pyseer using phylogeny-based distances, FaST-LMM implemented in pyseer using variant-based distances and dimensionality-reduction approach in SEER.
